# Supplementary figures and images for: Monkeys Predict US Elections
Source: bioRxiv. 2024 Sep 29:2024.09.17.613526. Originally published 2024 Sep 19. Preprint. [Version 2] doi: 10.1101/2024.09.17.613526 (PMC11429696; doi:10.1101/2024.09.17.613526)

# Supplementary Figure 1

**a**

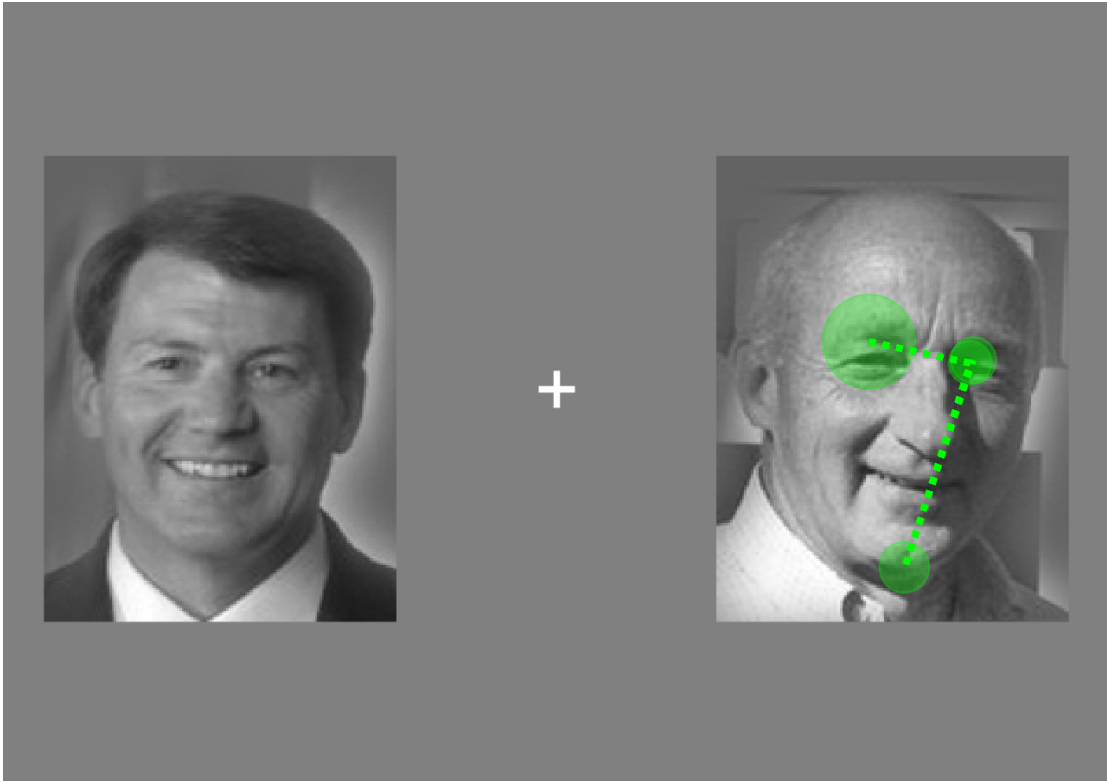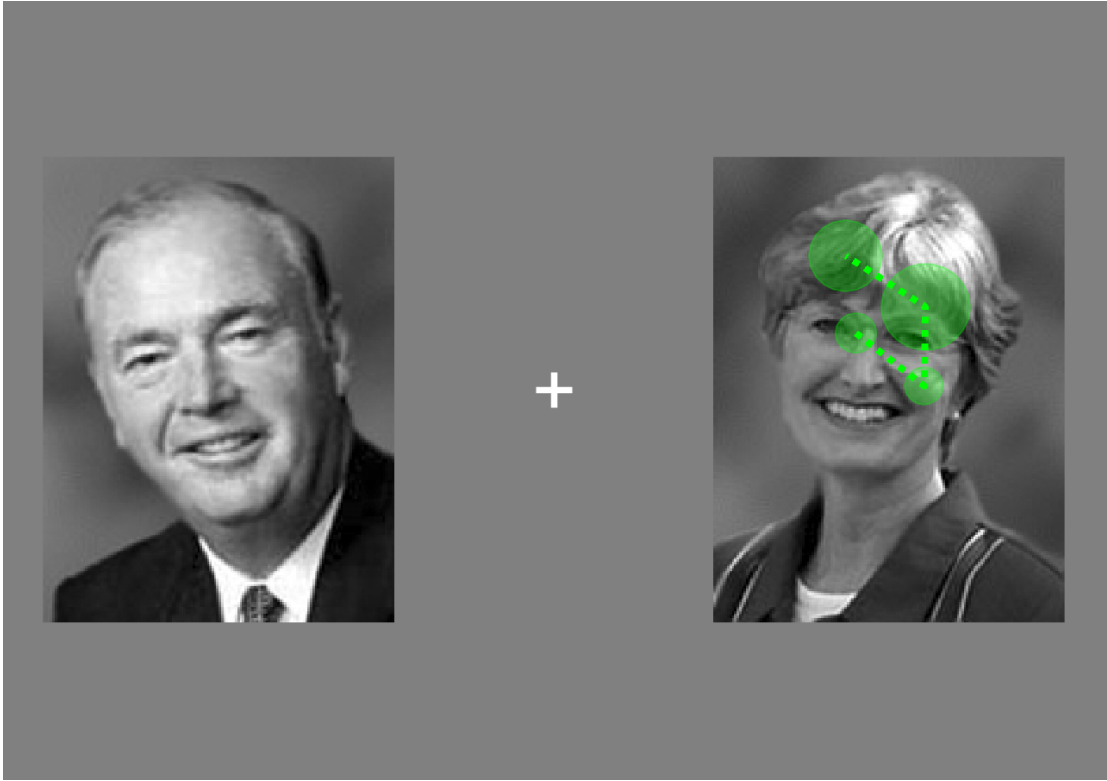

**b**

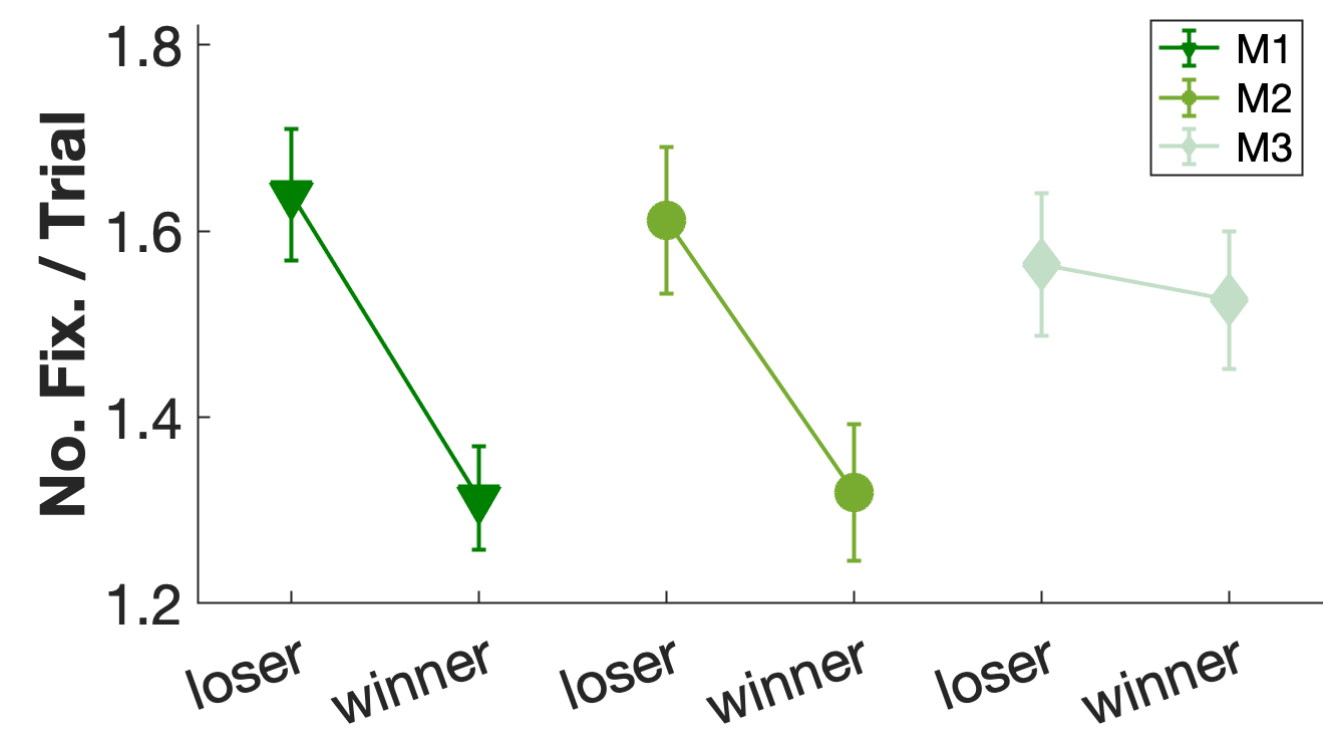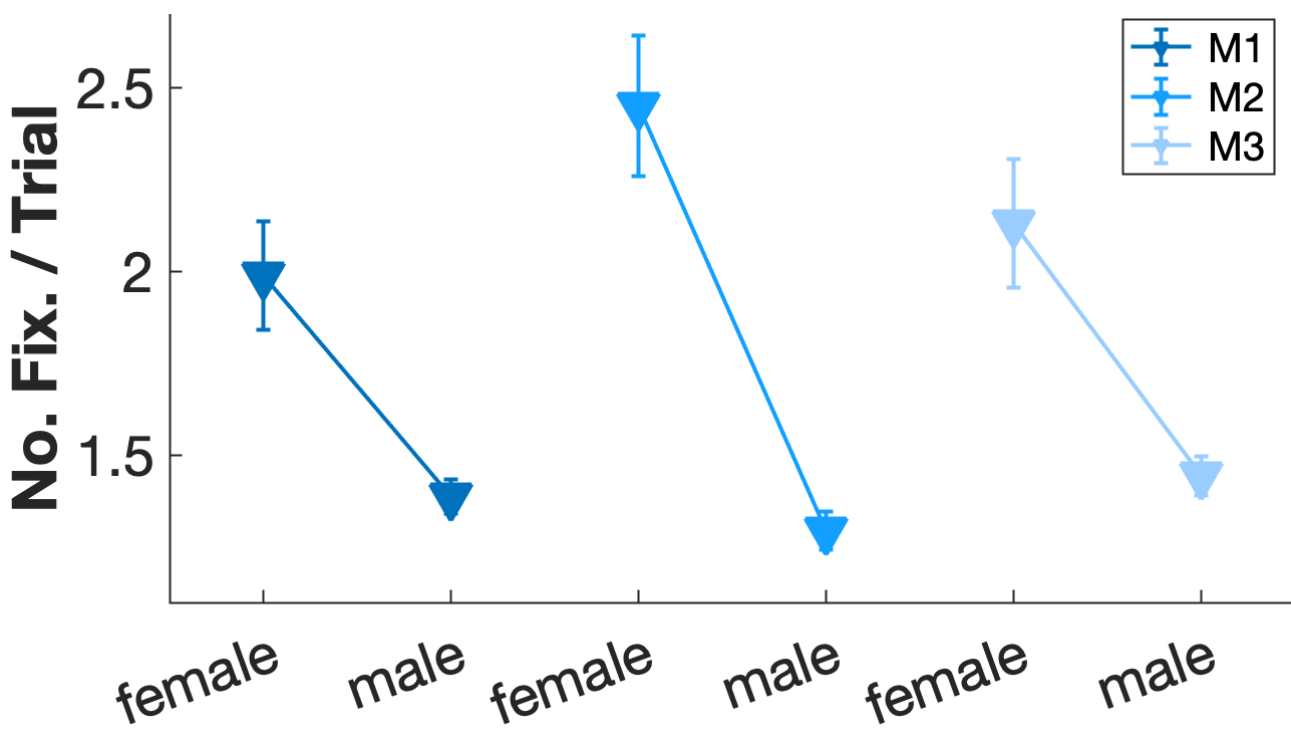

**c**

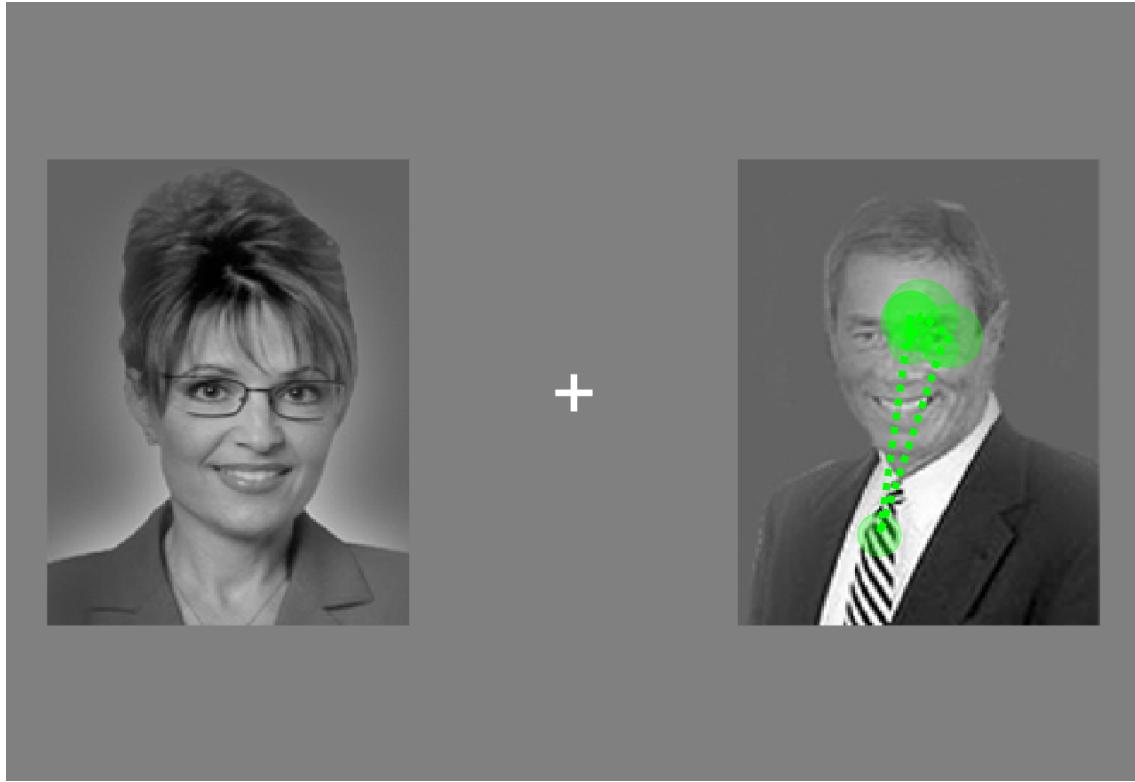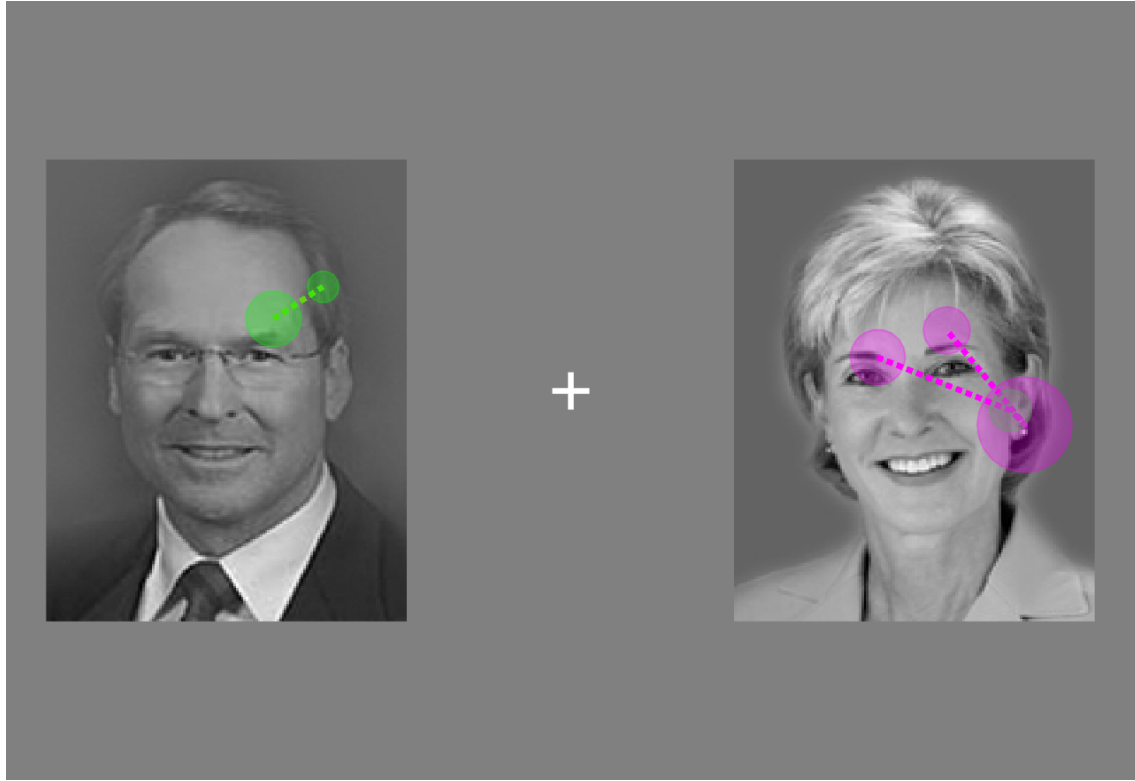

**d**

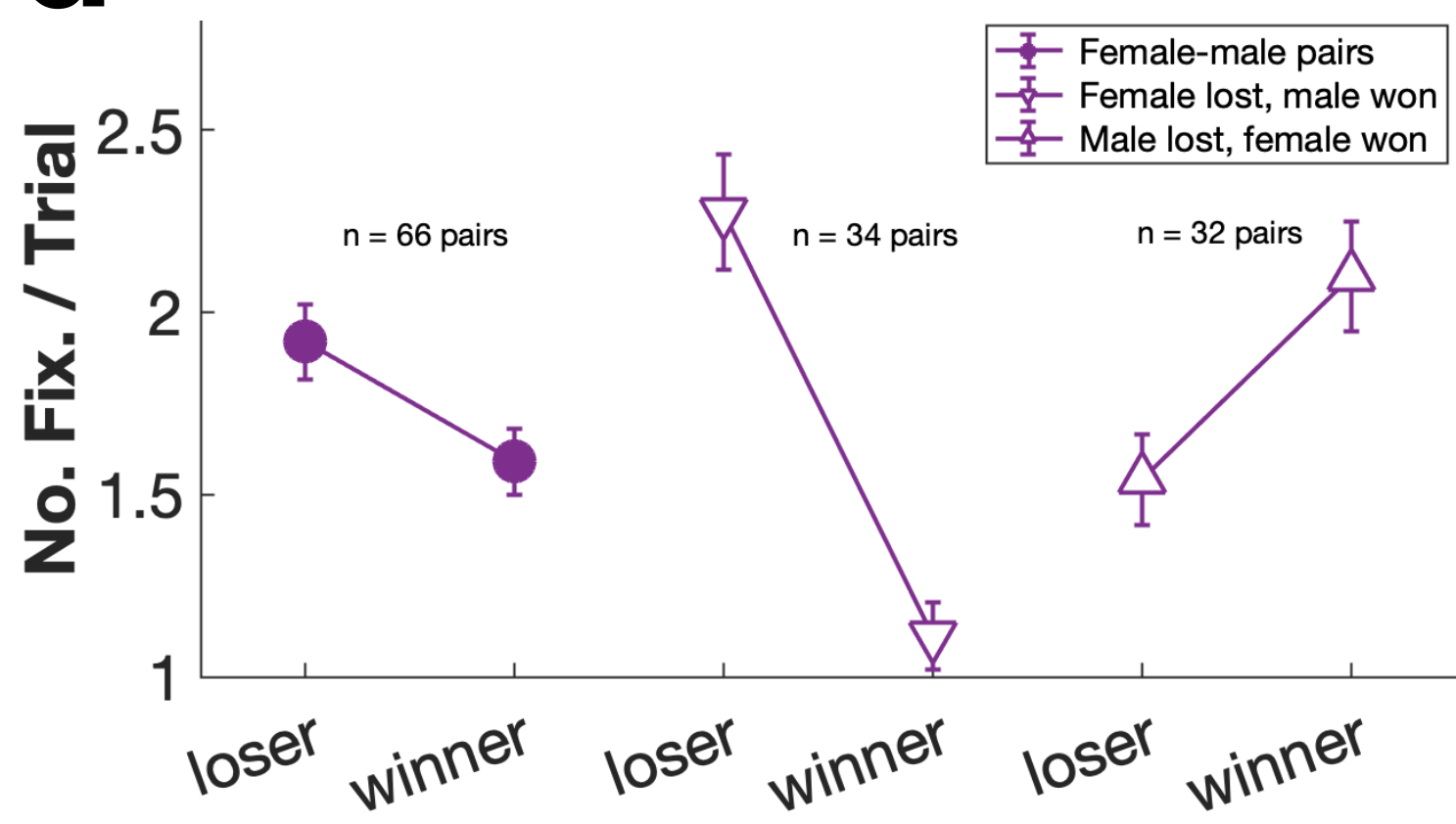

**e**

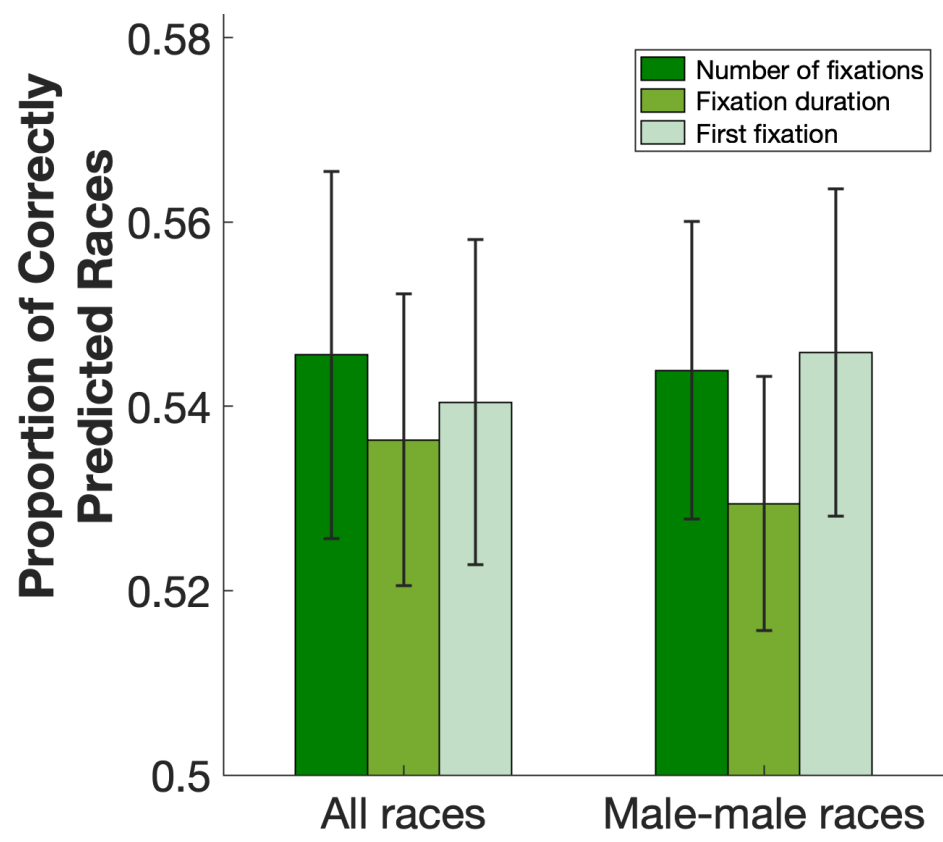

**f**

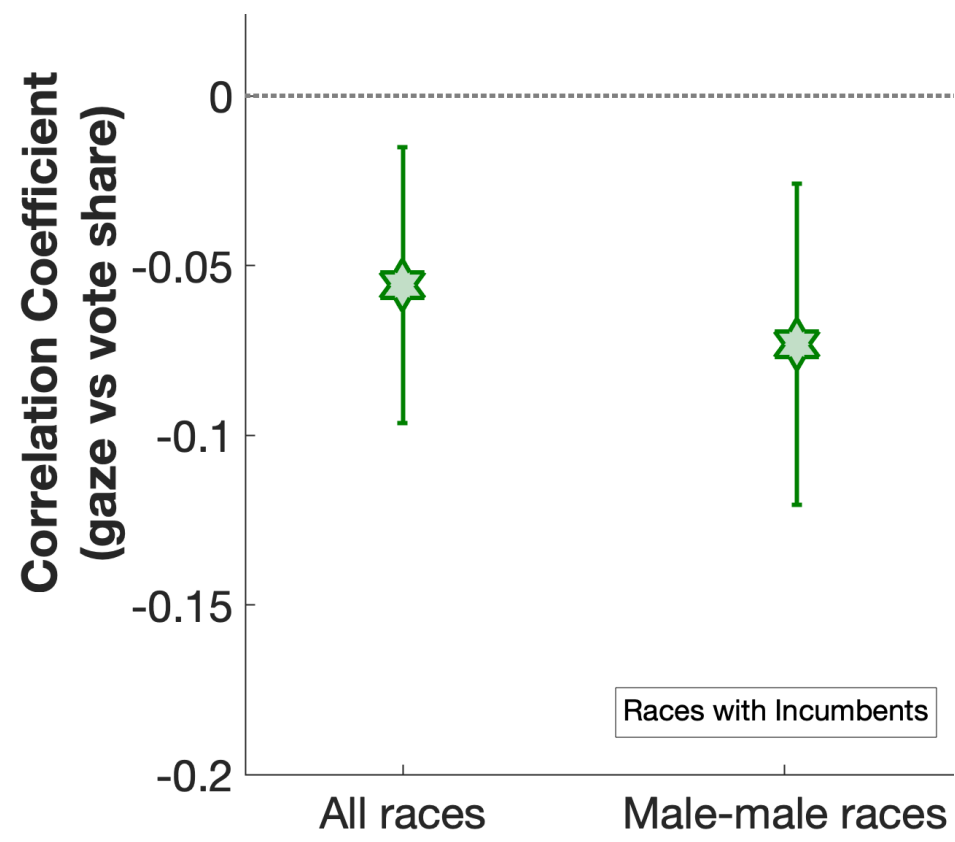

# Supplementary Figure 2

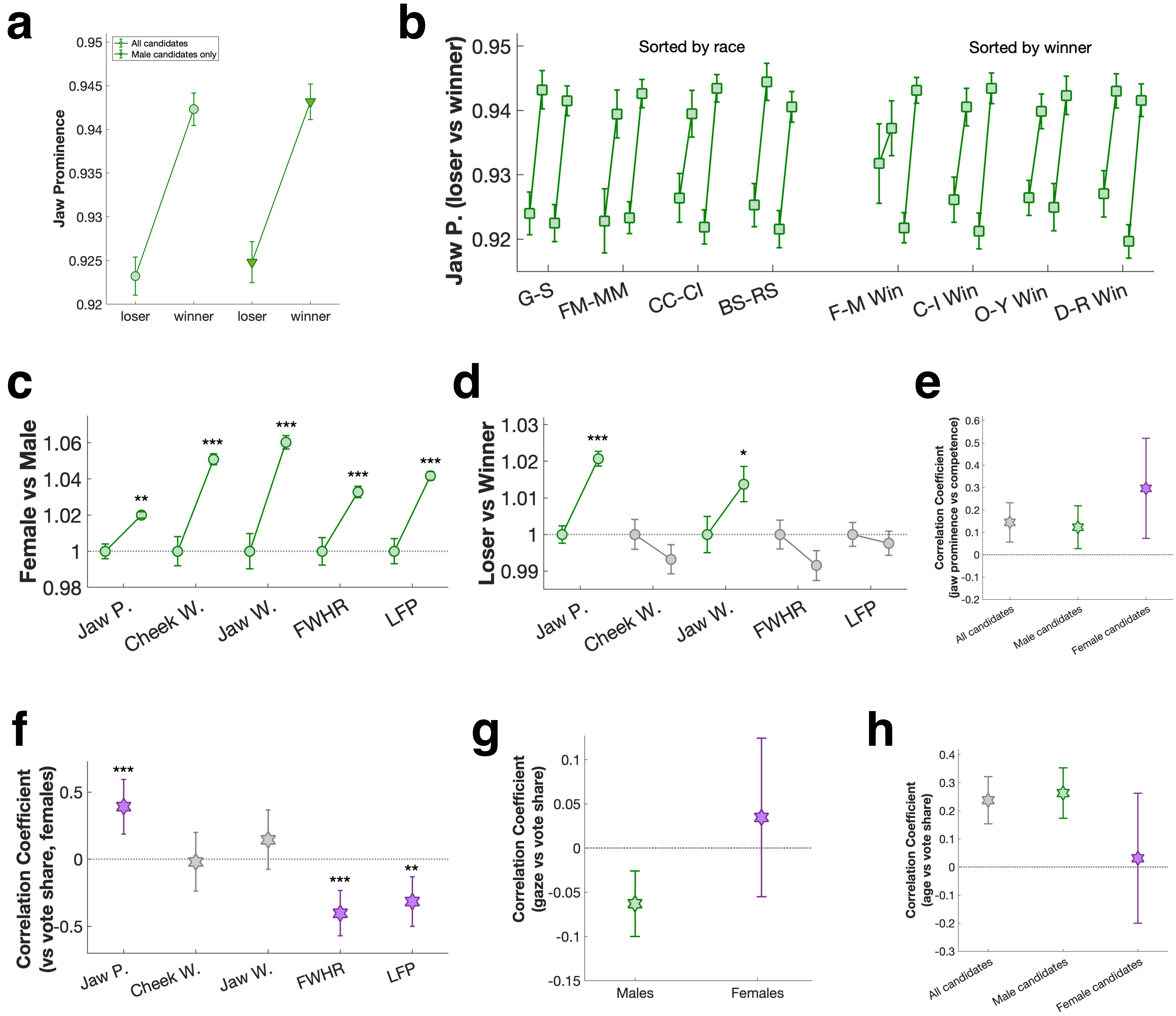

# Supplementary Figure 3

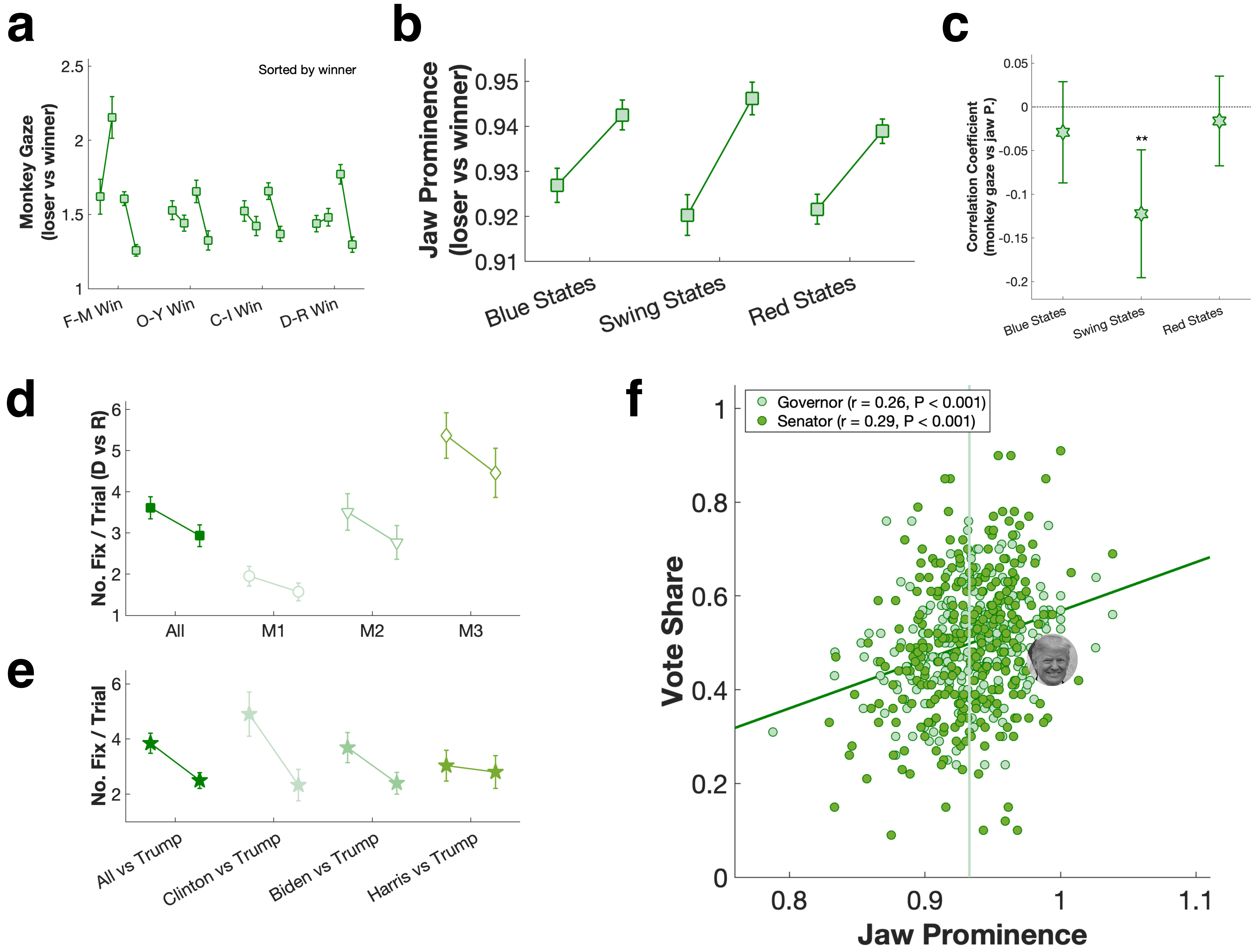

Supplement: Supplement 1 — Figure S1: a: Example gaze patterns for gubernatorial elections in South Dakota (2006, top) and Alaska (2002, bottom). Cross: central fixation spot. Filled circles: fixations on candidate pictures; circle size: fixation duration. Magenta: fixations on election winner; green: fixations on loser. b: Gaze bias for all 3 male monkeys as a function of election outcome (top), and candidate gender (bottom). Error bars: mean ± SEM. c: Example gaze patterns in races female candidate prevailed over male opponent for gubernatorial elections in Alaska (2002, top) and Kansas (2006, bottom). Cross: central fixation spot. Filled circles: fixations on candidate pictures; circle size: fixation duration. Magenta: fixations on election winner; green: fixations on loser. d: Gaze bias as a function of election outcome for female vs male races. Error bars: mean ± SEM. e: Gaze bias election predictions across analytical approaches. f: Correlation between gaze bias and vote share restricted to races with incumbents. Error bars: 95% confidence interval. Figure S2: a: Jawline bias as a function of election outcome. b: Jawline election outcome bias (loser vs. winner) as a function of election type (left) or winner identity (right). G-S: Gubernatorial and Senatorial races; FM-MM: Female-Male and Male-Male races; CC-CI: Challenger-Challenger and Challenger-Incumbent races; BS-RS: Blue State and Red State races; F-M win: Female-won and Male-won races; C-I win: Challenger-won and Incumbent-won races; O-Y win: Older-candidate-won and Younger-candidate-won races; D-R win: Democrat-won and Republican-won races. c: All facial masculinity cues as a function of candidate gender. d: All facial masculinity cues as a function of election outcome. Error bars: mean ± SEM. e: Correlation of jaw prominence with vote share for both male and female candidates. f: Correlations between all facial masculinity cues and vote share for female candidates. g: Correlation of monkey gaze bias with vote share as a functio [file media-1.pdf]
